# Complex Evolution of the Mismatch Repair System in Eukaryotes is Illuminated by Novel Archaeal Genomes

**DOI:** 10.1007/s00239-020-09979-5

**Published:** 2021-01-07

**Authors:** Paulo G. Hofstatter, Daniel J. G. Lahr

**Affiliations:** 1 Instituto de Biociências, Universidade de São Paulo (USP), Rua do Matão, trav. 14, A101, CEP., São Paulo, 05508-090 Brazil; 2 Max-Planck-Institut für Planzenzüchtungsforschung, Carl-von-Linné-Weg 10, 50829 Cologne, Germany

**Keywords:** Asgard Archaea, DNA repair, Eukaryotes, Mismatch repair, *mutL*, *mutS*

## Abstract

Repairing DNA damage is one of the most important functions of the ‘housekeeping’ proteins, as DNA molecules are constantly subject to different kinds of damage. An important mechanism of DNA repair is the mismatch repair system (MMR). In eukaryotes, it is more complex than it is in bacteria or Archaea due to an inflated number of paralogues produced as a result of an extensive process of gene duplication and further specialization upon the evolution of the first eukaryotes, including an important part of the meiotic machinery. Recently, the discovery and sequencing of Asgard Archaea allowed us to revisit the MMR system evolution with the addition of new data from a group that is closely related to the eukaryotic ancestor. This new analysis provided evidence for a complex evolutionary history of eukaryotic MMR: an archaeal origin for the nuclear MMR system in eukaryotes, with subsequent acquisitions of other MMR systems from organelles.

**Supplementary Information:**

The online version of this article (10.1007/s00239-020-09979-5) contains supplementary material, which is available to authorized users.

## Introduction

The DNA is relatively unstable and subject to frequent breaks and several kinds of damage. DNA damage may be a result of different physical and chemical agents, including UV radiation, gamma rays, free oxygen radicals, replication errors, among others (Lindahl 1993). The consequences of DNA damage range from point mutations to impairment of DNA function, compromising RNA transcription and protein synthesis, sometimes leading to cellular failure and death (Jackson and Bartek 2009). The evolution of DNA repair machineries allowed for efficient and specific DNA repair according to damage type and rescue of genetic functions. Such machineries became a fundamental part of cellular housekeeping genes. For each kind of damage, there is a specific repair system (Sancar et al. 2004). The well-characterized DNA repair systems so far include homologous recombination (Seitz et al. 1998), photolyases (Essen and Klar 2006), base excision repair (Krokan and Bjørås 2013), nucleotide excision repair (Schärer 2013), non-homologous end joining (Chang et al. 2017), mismatch repair (Li 2008) and polymerases (Wood and Shivji 1997). Most of them are widely distributed in all cellular domains (Bacteria, Archaea and Eukarya) and bear a high level of primary sequence conservation, as in the case of the recombinases (*recA* superfamily) implied in homologous recombination and *mutS*–*mutL* involved in mismatch repair (MMR) system (Lin et al. 2006, 2007).

Eukaryotic recombinases have been proposed to be related to Archaeal recombinases, which underwent ancestral duplication events in order to provide the several eukaryotic paralogues (Lin et al. 2006), one of them a component of the meiosis-specific machinery (Bishop et al. 1992). There is a second kind of recombinase in eukaryotes, this one with a bacterial signature. In this case, their origin can be traced back to both Alpha-proteobacteria and Cyanobacteria. They are a result of endosymbiotic gene transfers from mitochondria and chloroplasts to eukaryotes and because they possess a targeting domain, are thought to be active only inside endosymbiotic organelles (Hofstatter et al. 2016). Similarly, the eukaryotic MMR system proteins MutS and MutL homologues evolved by means of a series of gene duplications yielding several paralogues in the ancestral of eukaryotes (Lin et al. 2007), some of which present meiosis-specific functions as well (Hollingsworth et al. 1995). However, differently from the recombinases, the MutS–MutL system implied in nuclear MMR in eukaryotes has been proposed to be a contribution from the mitochondrial ancestor to early eukaryotes (Lin et al. 2007). Interestingly, bacterial *mutS* and *mutL* occur in some Archaeal groups as well, probably as a result of horizontal gene transfers (HGT) from Bacteria to Archaea (Lin et al. 2007). Recent evidence further suggests that two kinds of MMR systems evolved independently in the ancestors of both Bacteria and Archaea after their divergence, the *NucS* system in Archaea and *mutS*–*mutL* system in Bacteria with some level of exchange of these genes later (Castañeda-García et al. 2017; Takemoto et al. 2018). The MutS dimer works together with MutL dimer in order to perform DNA repair. A dimer of MutS proteins identifies and is attracted to a site of mismatch on a DNA molecule and recruits a dimer of MutL, which cuts and removes the mismatch, allowing the action of DNA polymerases and DNA ligases to fill the gap and finish the process (Modrich and Lahue 1996). Some bacterial groups (e.g. *Escherichia coli*) also present MutH, which bias the nicking and removing the mismatched site towards the unmethylated strand, i.e. the newly synthesized one (Smith and Modrich 1996). Although the system bears a clear archaeal signature, *NucS* occurs in Actinobacteria, probably due to an early HGT from Archaea to this bacterial group. In addition, both systems may occur in some archaeal groups (Castañeda-García et al. 2017). Even though archaeal in nature, *NucS* homologues do not occur in eukaryotes and were probably lost in the transition from Archaea to the first eukaryotes.

The bacterial MMR system composed of *mutS* and *mutL* was acquired by eukaryotes and ancestrally duplicated several times producing the eukaryotic paralogues that occur in most eukaryotic lineages (Modrich and Lahue 1996; Lin et al. 2007). Six mutS eukaryotic paralogues are widespread in eukaryotes, namely *MSH1*-*6*; additionally, there are four *mutL* paralogues, namely *MLH1-4*. All of them interact in specific ways with each other. Paralogues *MSH4* and *MSH5* are meiosis-specific and do not realize MMR anymore, but participate of crossing-over resolution during meiosis (Hollingsworth et al. 1995). The evolution of MMR in eukaryotes is linked to the evolution of meiosis itself. All the eukaryotic *mutS* homologues are highly conserved and operate MMR not only inside the nucleus, but also inside organelles as well. The activity of *mutS* homologues inside organelles, the bacterial signature of this DNA repair system, and phylogenetic patterns observed by Lin and colleagues (Lin et al. 2007) led to the assumption that the whole eukaryotic MMR system was acquired by eukaryotes laterally from Alpha-proteobacteria upon the mitochondrial endosymbiotic event. However, the discovery of a new group of Archaea closely related to eukaryotes, the Asgard Archaea, offered the opportunity to revisit this matter (Zaremba-Niedzwiedzka et al. 2017). In this study, we revisit the subject with more comprehensive data and more advanced techniques in order to verify the putative mitochondrial contribution to meiosis in eukaryotes.

## Results

**Fig. 1:**
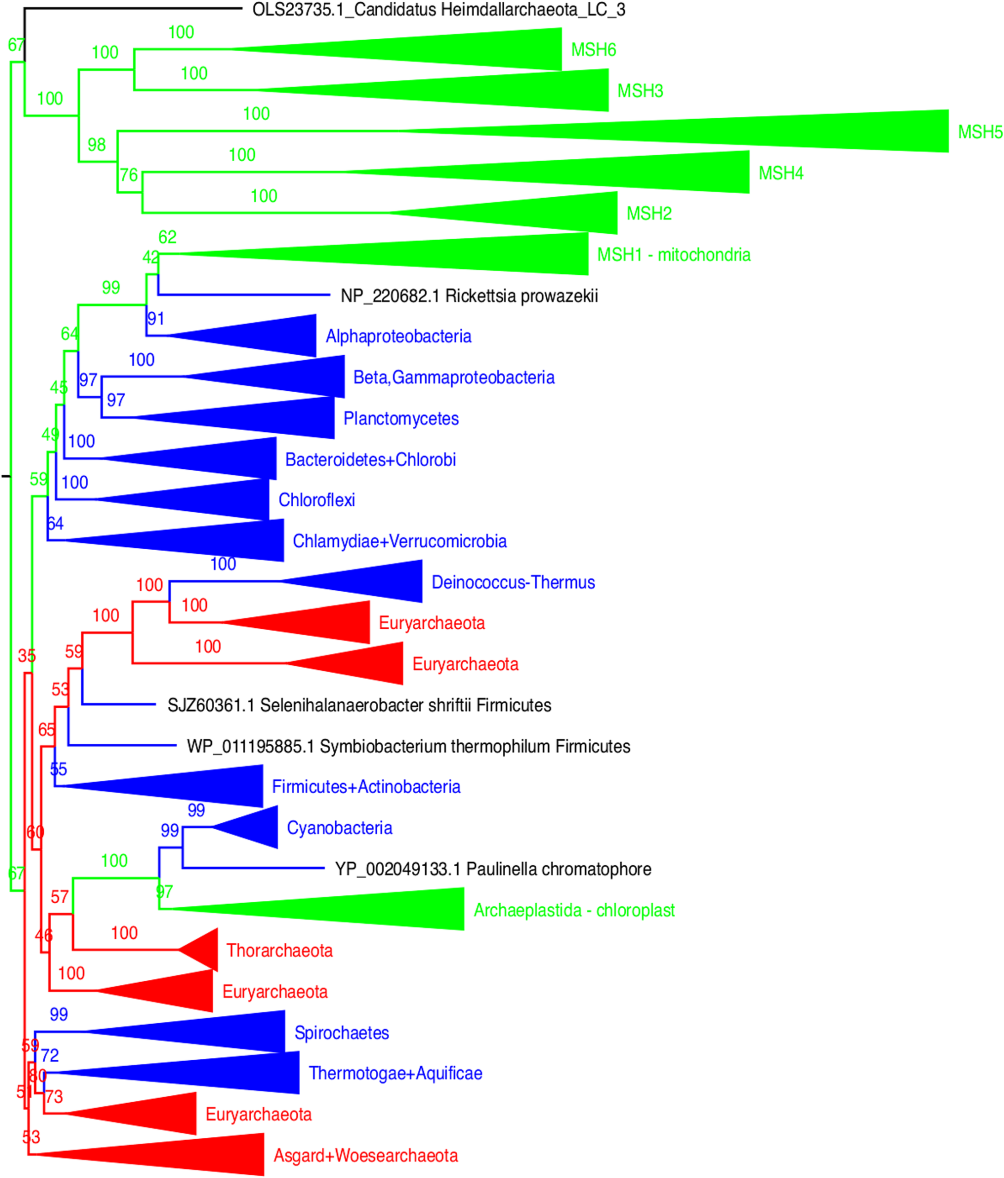
mutS protein tree. The eukaryotic homologues of bacterial mutS have three different sources: one associated with Asgard Archaea (nuclear *mutS* homologues *MSH2*, *MSH3*, *MSH4*, *MSH5*, *MSH6*, *MSH7*), one associated with Alpha-proteobacteria (mitochondrial *MSH1*), and another one associated with Cyanobacteria (chloroplastic *MSH1*)

Contrary to previous conclusions (Lin et al. 2007), the main group of eukaryotic *mutS* paralogues active inside the nucleus (paralogues *MSH2-6*) are not a mitochondrial contribution but are a result of gene duplications of a *mutS* gene present in the archaeal ancestor. The archaeal ancestor in turn, probably acquired the *mutS* laterally from a bacterial group very long ago, before the evolution of the first eukaryotes (Fig. 1, Suppl. Tree 1). In Asgard Archaea, *mutS* and *mutL* occur *in tandem* in the same way they occur in Firmicutes. This fact makes Firmicutes a good candidate for a donor of the bacterial MMR to Asgard group. The same reconstructions reveal the mitochondrial origin of eukaryotic *MSH1*, which has an Alpha-proteobacterial signature. Still in the same reconstructions, there is a third group of eukaryotic *mutS* with cyanobacterial origin, acquired from the chloroplast ancestor in some photosynthesizing groups.

**Fig. 2:**
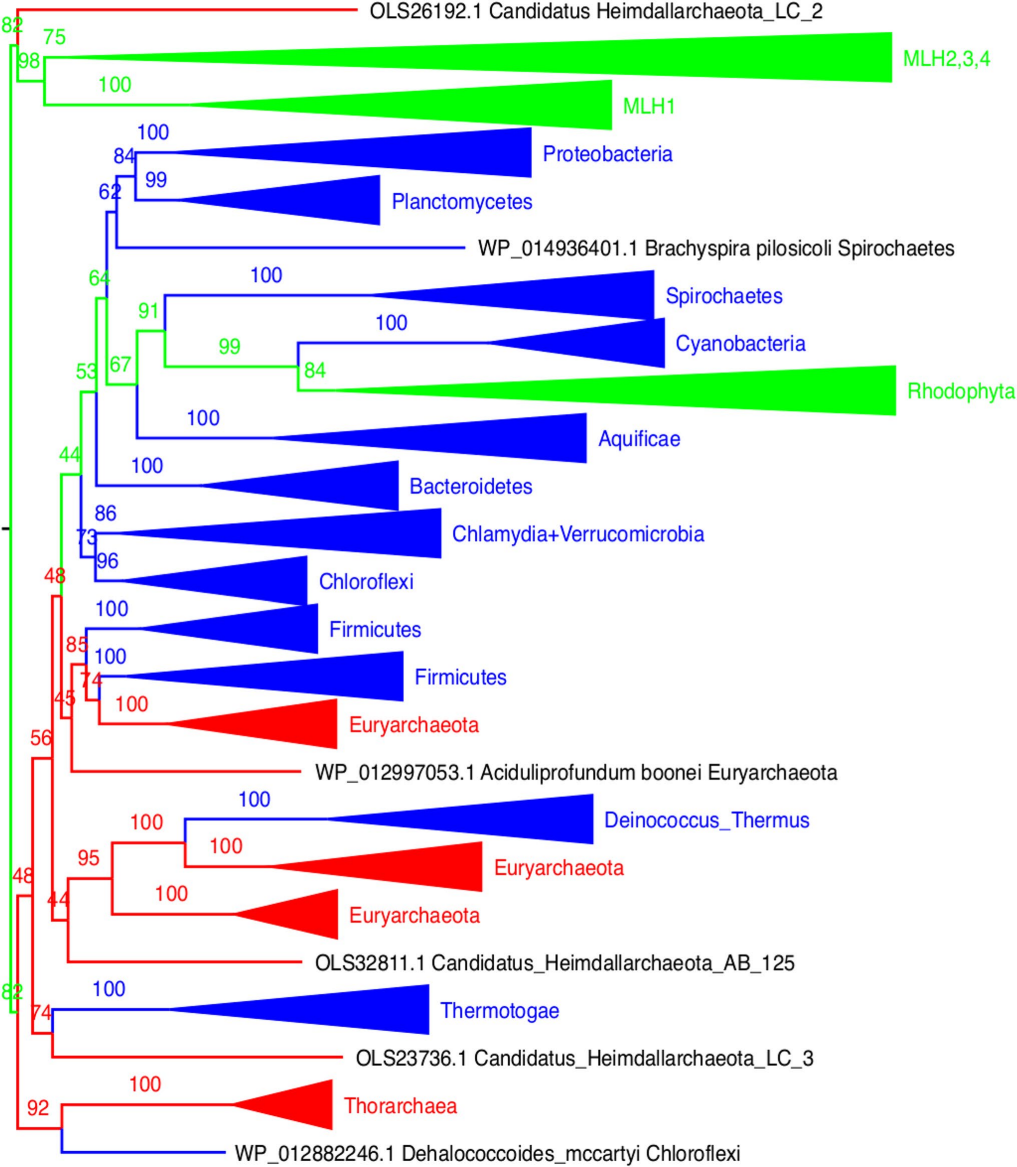
mutL protein tree. The eukaryotic *mutL* homologues have two distinguishable sources: the nuclear *mutL* homologues (*MLH1*, *MLH3*, *MLH3*, *MLH4*) are associated with Asgard Archaea, and another branch of eukaryotic *mutL* are associated with Cyanobacteria (chloroplast). No mitochondria-associated MutL proteins could be traced back to Alpha-proteobacteria

The same applies to *mutL*: *mutS* and *mutL* were probably acquired together by the archaeal group that gave origin to eukaryotes and then both underwent gene duplications that are an important step in early eukaryotic evolution. Systematic reconstructions of both *mutS* and *mutL* proteins exhibit similar patterns. Patterns observed in the *mutL* reconstructions are similar to the ones observed in the case of *mutS* (Fig. 2, Supple. Tree 2). The nuclear paralogues *MLH1-4* exhibit the same patterns of the nuclear *MHS2-6*. Both *mutS* and *mutL* must have been acquired together, what are expected as both work together as part of the same complex. The mitochondrial *mutL* seems to have been lost and probably replaced by some nuclear paralogue. The chloroplastic *mutL* was seemingly lost in the green algae, but kept by red algae. A concatenation of *mutS* and *mutL* provides a similar result regarding the evolution of both proteins when treated alone, but at this time, all Heimdallarchaea are attracted towards the nuclear eukaryotic group (Suppl. Fig. 1, Suppl. Tree 3). This may be interpreted as an increase in the signal of the reconstruction, assuming both genes evolved in concerted evolution.

## Discussion

The importance of the MMR repair for maintenance of the integrity of the DNA and the high level of conservation of its components across all cellular domains have raised questions about the evolution of the whole MMR system, specially in eukaryotes (Lin et al. 2007). However, by revisiting this matter, we have found a very different history concerning *mutS* and *mutL* evolution. Back then, a mitochondrial origin for all eukaryotic paralogues was proposed, including both nuclear and mitochondrial forms. The chloroplastic ones could not be distinguished with those data. The introduction of Asgard data was crucial for retelling this history. Asgard is an archaeal group closely related to eukaryotes, or at least, is the closest known archaeal group to eukaryotes that was sampled to this day (Zaremba-Niedzwiedzka et al. 2017).

**Fig. 3:**
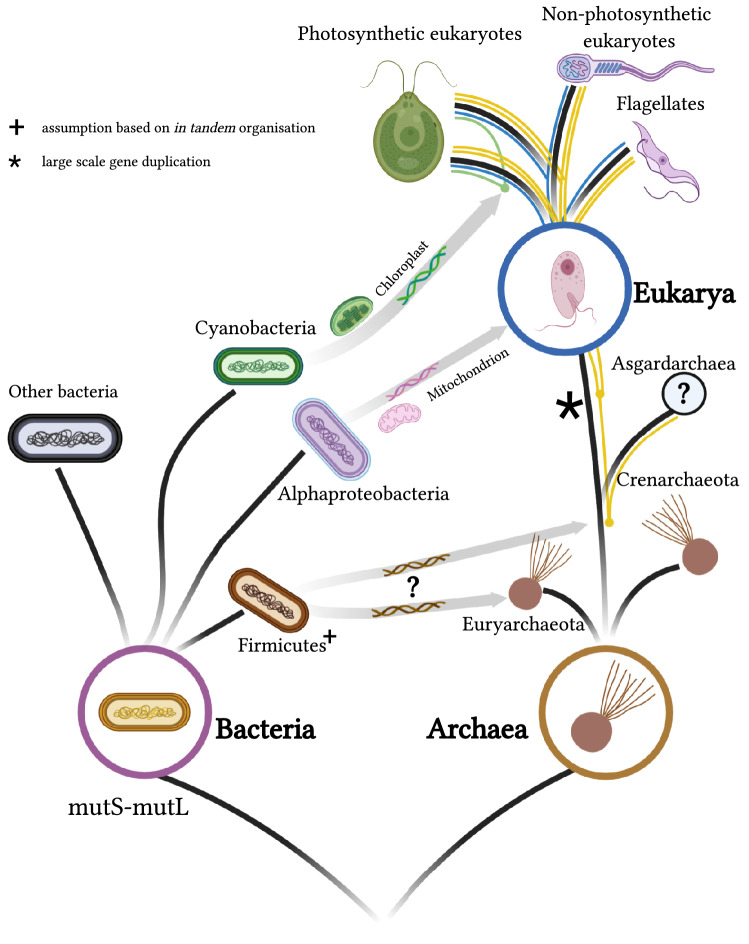
Main events in the evolution of mismatch repair machinery composed by *mutS*–*mutL* system: Eukaryotic *mutS* and *mutL* were inherited vertically from archaeal ancestors and then acquired laterally twice upon endosymbiotic events. The transition from Archaea to eukaryotes was characterized by large-scale duplication events to produce all the nuclear paralogues; later, an acquisition from the mitochondrial ancestor, which is encoded in the nuclear genome, but is active inside the organelles, and again from the chloroplast in the photosynthesizing eukaryotes, also encoded in the nuclear genome, but active inside de organelles. Heimdallarchaeota is sister group to all eukaryotic nuclear paralogues. Archaeal groups themselves seem to have laterally acquired the *mutS*–*mutL* system from some bacterial donor once or more

Eukaryotic *mutS* homologues were acquired three times independently by eukaryotes: one vertically from archaeal ancestors, then laterally from the mitochondrial ancestor, and again laterally from the chloroplastic ancestor in the photosynthesizing lineages (Fig. 3).

The discovery that the nuclear MMR duties are carried out in eukaryotes by proteins of archaeal origin is more plausible than a mitochondrial origin for this process because other kinds of DNA repair systems that occur in eukaryotes are also of archaeal origin (Lin et al. 2006; Malik et al. 2007). Not only DNA repair systems, but also eukaryotic DNA replication and transcription machineries are of archaeal origin (Kwapisz et al. 2008; Makarova et al. 2014). Contributions of the archaeal ancestor tend to be ‘informational’, while mitochondrial (and chloroplastic) contributions tend to be ‘operational’ in nature (Rivera et al. 1998). Proteins active inside organelles that are synthesized in the cell cytoplasm and imported by specialized mechanisms into mitochondria and chloroplasts (Wiedemann et al. 2004). Mitochondria and chloroplasts import the products of genes that were endosymbiotically transferred to the nuclear genome by means of a system of transit peptides that precede active sites of the mature active proteins implied in the process (Neupert and Herrmann 2007). Earlier analyses suggest a mitochondrial contribution of Alpha-proteobacteria to eukaryotic DNA repair system and meiosis (Lin et al. 2007); the new results support a very different history, which implies no mitochondrial contribution known so far to eukaryotic nuclear processes. In addition, the importance of the mitochondrial endosymbiont to meiosis was overestimated. As the mitochondrial variable seems to have been isolated here, the understanding of the evolution of meiosis may be developed independently from the mitochondrial symbiosis event. In this scenario, sex itself may be older than the mitochondrion in eukaryotes. The discovery of the Asgard Archaea group played a fundamental role at establishing a new version for the evolutionary history of the MMR in eukaryotes.

## Material and Methods

A strategic sampling of *mutS* and *mutL* representatives was performed in order to maximize the number of bacterial and archaeal phyla in the sampling, while only a few eukaryotic representatives were selected. The mismatch repair system based on MutS–MutL proteins is almost ubiquitous in Eubacteria, except for Actinobacteria, where it was seemingly replaced by *NucS* (Castañeda-García et al. 2017). Thus, a handful of representatives of every major bacterial phylum was included. Preference was given to well-characterized species with whole genome sequenced of Proteobateria, Cyanobacteria, Chloroflexi, Chlamydia, Deinococcus, Firmicutes, Thermotogae and Spirochaetes, which account for the majority of extant bacterial lineages and include the most probable ancestors of eukaryotic organelles (mitochondria belong to Alpha-proteobacteria and chloroplasts belong to Cyanobacteria). A similar approach was applied to the case of Archaea with a special attention given to the Asgard Archaea group because of its recently proposed close relationship to eukaryotes (Zaremba-Niedzwiedzka et al. 2017). In the case of eukaryotes, very distantly related eukaryotic representatives have been selected in order to represent a less biased sampling of the group: *Octopus* (Metazoa), *Arabidopsis* (green plants), *Saccharomyces* (Fungi), *Dictyostelium* (Amoebozoa) and *Monocercomonoides* (a flagellated protist assumed to be early branching among eukaryotes).

For the construction of HMMER profiles, we employed sequences from mismatch repair obtained and characterized from eukaryotic model organisms, such as *Saccharomyces*, *Arabidopsis* or *Homo.* They were used as a starting point. Different eukaryotic paralogues were aligned with 100 iterations of MAFFT (Katoh and Standley 2013) aligner for precision. The aligned set of proteins was given as input for HMMER tool *hmmbuild (*Eddy 2011*)*, which was employed to build both MutS and MutL profiles used in the searches. Profiles were employed by HMMER tool *hmmsearch* (Eddy 2011) to search in the databases (built with strategic sampling) for MutS and MutL. Best hits were extracted from databases with HMMER tool *esl-sfetch* (Eddy 2011) for further processing. Again, resulting sequences were aligned with 100 iterations of MAFFT (Katoh and Standley 2013); the resulting matrix trimmed with Trimal (Capella-Gutiérrez et al. 2009) where the sites had more than 50% indels/unaligned positions. IQ-Tree (Nguyen et al. 2015) was chosen as a state-of-art algorithm for reconstructions. Heavy mixture models (LG + C60 + F + G) were set for reconstructions for both MutS- and MutL-trimmed matrices. Standard number of bootstrap replicates (1000) was applied. An additional concatenated MutS–MutL matrix was produced for a third tree (in this case, eukaryotic MLH1 and MSH6 were arbitrarily chosen). In addition, Asgard data were manually checked to verify the relative position of *mutS* and *mutL* homologues in relation to each other in the available Asgard contigs.

## Supplementary Information

Below is the link to the electronic supplementary material.

(hmm 363 kb)

(hmm 358 kb)

(trim 68 kb)

(contree 11 kb)

(txt 11 kb)

(trim 152 kb)

(contree 11 kb)

(trim 102 kb)
